# A malignant solitary fibrous tumour arising from the first lumbar vertebra and mimicking an osteosarcoma: a case report

**DOI:** 10.1186/s12957-017-1161-0

**Published:** 2017-05-11

**Authors:** Naoki Oike, Hiroyuki Kawashima, Akira Ogose, Tetsuo Hotta, Toru Hirano, Takashi Ariizumi, Tetsuro Yamagishi, Hajime Umezu, Shoichi Inagawa, Naoto Endo

**Affiliations:** 1 Division of Orthopedic Surgery, Graduate School of Medical and Dental Sciences, Niigata University, 1-757 Asahimachi-dori, Niigata, 951-8510 Japan; 2 Division of Pathology, Graduate School of Medical and Dental Sciences, Niigata University, Niigata, Japan; 3 Division of Radiology, Graduate School of Medical and Dental Sciences, Niigata University, Niigata, Japan

**Keywords:** Malignant solitary fibrous tumour, Osteosarcoma, Lumbar vertebra, Anterior spinal fusion, Fusion gene

## Abstract

**Background:**

A solitary fibrous tumour (SFT) is an unusual neoplasm typically found in soft tissues. Although SFTs can arise in the bones, they very rarely arise in the vertebral arch. Here, we describe a case of a SFT that arose in the vertebral arch of the first lumbar (L1) spinal vertebrae and mimicked osteosarcoma.

**Case presentation:**

A 49-year-old woman presented with a 2-month history of lower back pain and a lumbar region mass. Magnetic resonance imaging demonstrated a heterogeneously enhanced mass in the L1 vertebral arch. The patient received neoadjuvant chemotherapy, followed by a surgical procedure comprising an anterior spinal fusion and en bloc resection. Histologically, our initial diagnosis was osteosarcoma. The postoperative course was uneventful, and the patient received adjuvant chemotherapy. However, the tumour metastasised to the lung 5 years after the first surgery, and a second surgery was performed for lung tumour resection. The histology of the metastatic lung tumour appeared similar to that of the malignant SFT, and the specimen from the first surgery was re-examined. Immunohistochemically, the tumour was positive for STAT6. Reverse transcription-polymerase chain reaction revealed a *NAB2-STAT6* fusion gene, thus confirming our final diagnosis of malignant SFT. The patient died of disease progression 8 years after the first surgery; however, there was no evidence of local recurrence.

**Conclusions:**

Malignant SFT in the vertebral arch is extremely rare and very difficult to distinguish histologically an osteoid from lace-like collagen. STAT6 immunostaining is useful for distinguishing malignant SFTs from other neoplasms. Although it is difficult to completely resect a SFT arising from the spine, we demonstrated the feasibility of an en bloc resection of spinal tumours arising from posterior elements, without local recurrence.

## Background

A solitary fibrous tumour (SFT) is a rare type of spindle cell tumour that was first described to originate from the pleura [1]. Although SFTs mainly occur in the pleura, these tumours have been recently found to arise at various extrapleural sites, including the bone [2–4]. However, to the best of our knowledge, no reports have described malignant SFTs arising in the vertebral arch. Furthermore, it is extremely difficult to histologically distinguish malignant SFTs from other types of neoplasm, especially osteosarcoma. In this report, we describe a malignant case of a SFT that arose in the vertebral arch of the first lumbar (L1) vertebra and was initially diagnosed as an osteosarcoma.

## Case presentation

### History and examination

**Fig. 1:**
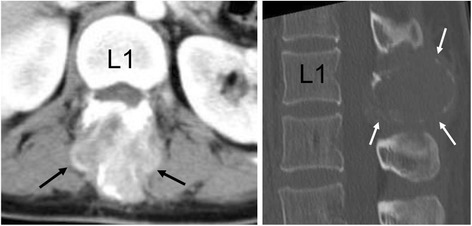
Preoperative computed tomography (CT). *Left*: Axial contrast-enhanced CT image reveals a heterogeneously enhanced mass (*arrows*) in the L1 vertebra. *Right*: Sagittal reconstructed bone window CT image of the lumbar spine shows an expansile lytic lesion (*arrows*) in the posterior L1 vertebral arch. Abbreviation: *L1* body of the first lumbar vertebra

**Fig. 2:**
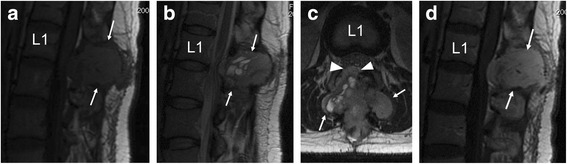
Preoperative magnetic resonance (MR) imaging. **a** Sagittal T1-weighted MR image shows an extradural tumour exhibiting mild hyperintensity in the L1 vertebral arch (*arrows*). **b** Sagittal T2-weighted MR image shows a mass with heterogeneous hyperintensity (*arrows*). **c** Axial T2-weighted image reveals a posterior epidural component of the L1 arch lesion (*arrows*), with compression of the thecal sac (*arrow heads*). d Sagittal gadolinium-enhanced MR image reveals a well-enhanced tumour in the L1 vertebral arch (*arrows*). Abbreviation: *L1* body of the first lumbar vertebra

A 49-year-old woman was referred to our hospital with a 2-month history of lower back pain and a mass in her lumbar region. The patient provided written informed consent for the use of her case data. Physical examination revealed a tender, immobile mass in her lumbar region, but no weakness or sensory deficits. Computed tomography (CT) revealed a 3 × 3 × 4 cm mass exhibiting inhomogeneous contrast enhancement in the vertebral arch of the L1 vertebra (Fig. 1, left), as well as a lytic lesion in the vertebral arch of the L1 vertebra (Fig. 1, right). Magnetic resonance imaging (MRI) showed an extradural tumour in the L1 vertebral arch with mild hyperintensity on T1-weighted imaging (Fig. 2a) and heterogeneously hyper- and hypointensity on T2-weighted imaging (Fig. 2b). Axial T2-weighted imaging demonstrated that the mass had compressed the spinal cord (Fig. 2c). Gadolinium-enhanced fat-suppressed T1-weighted images revealed a well-enhanced tumour that extended to the subcutaneous tissues at the L1 level (Fig. 2d). An open biopsy was performed to facilitate diagnosis, and specimen pathology indicated a type of undetermined malignant neoplasm. The patient received one course of chemotherapy comprising ifosfamide, doxorubicin, and etoposide.

### Operative procedure

A marginal resection and anterior spinal fusion were performed from the 12th thoracic (T12) to the third lumbar (L3) vertebra. During surgery, the patient was placed in the right lateral position. The extrapleural retroperitoneal approach was used, and the lateral aspect of the vertebral body was exposed from T12 to L3. Anterior vertebral screws were placed in the vertebral bodies from T12 to L3, and the disks and end-plates were removed. Subsequently, iliac bone and rib allografts were implanted, and rods were inserted using screws. After wound closure, the patient was placed in the prone position.

**Fig. 3:**
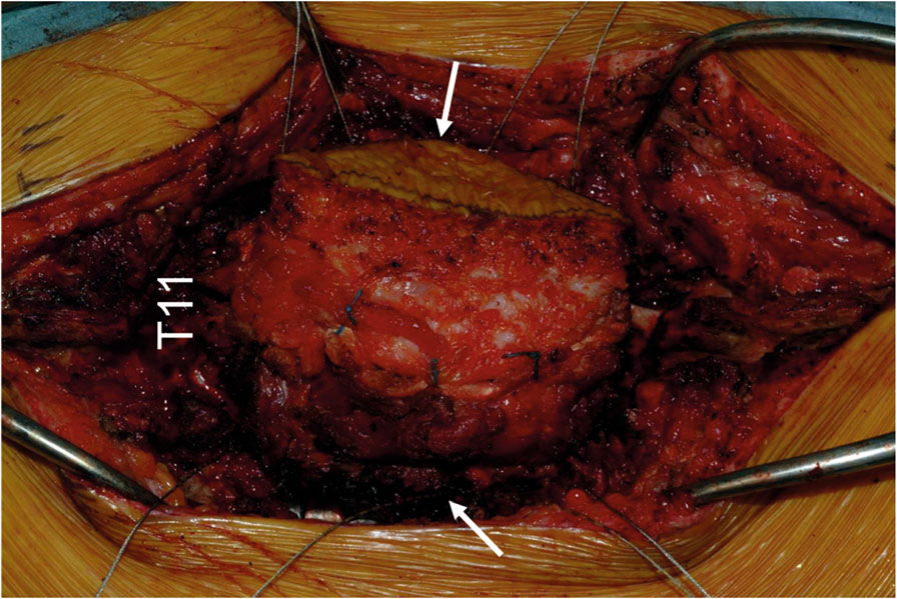
Intraoperative photography. The T-saw was inserted from the lateral L1/2 foramen (*arrows*) to the superior or inferior side of the spinal canal. Abbreviation: *T11* vertebral arch of the 11th thoracic vertebra

**Fig. 4:**
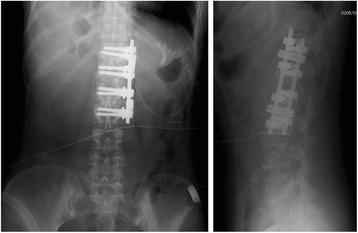
Postoperative radiographs. Postoperative anteroposterior (*left*) and lateral (*right*) radiographs of the spine show the anterior spinal fusion with instrumentation from T12 to L3

A lens-shaped incision that included the biopsy scar was made with a 2-cm margin from the tumour and deepened carefully to maintain a wide curative margin. The bilateral pars interarticularis of T12, tips of the transverse processes of L1 and L2, facet joint of L2/3, and vertebral arch of L3 were exposed. The bilateral pars interarticularis of T12 were cut using a high-speed drill, and a L2/3 facetectomy was performed. Elastic T-saw guide tubes were inserted into the spinal canal from the outside of the L1/2 neural foramen and was advanced cranially (to cut the L1 pedicle) or caudally (to cut the L2 pedicle). The T-saw was subsequently passed through the guide tube and successfully placed around the bilateral L1 and L2 pedicles (Fig. 3). Finally, the pedicles were cut, and the tumour was resected en bloc along with the posterior elements of T12 to L2 and the surrounding soft tissue (Fig. 4, left and right).

### Pathological findings

Macroscopically, the resected tumour measured 3.7 × 3.4 × 4.4 cm in diameter. The cut surface revealed a pale, solid mass with skeletal muscle and subcutaneous tissue infiltration.

**Fig. 5:**
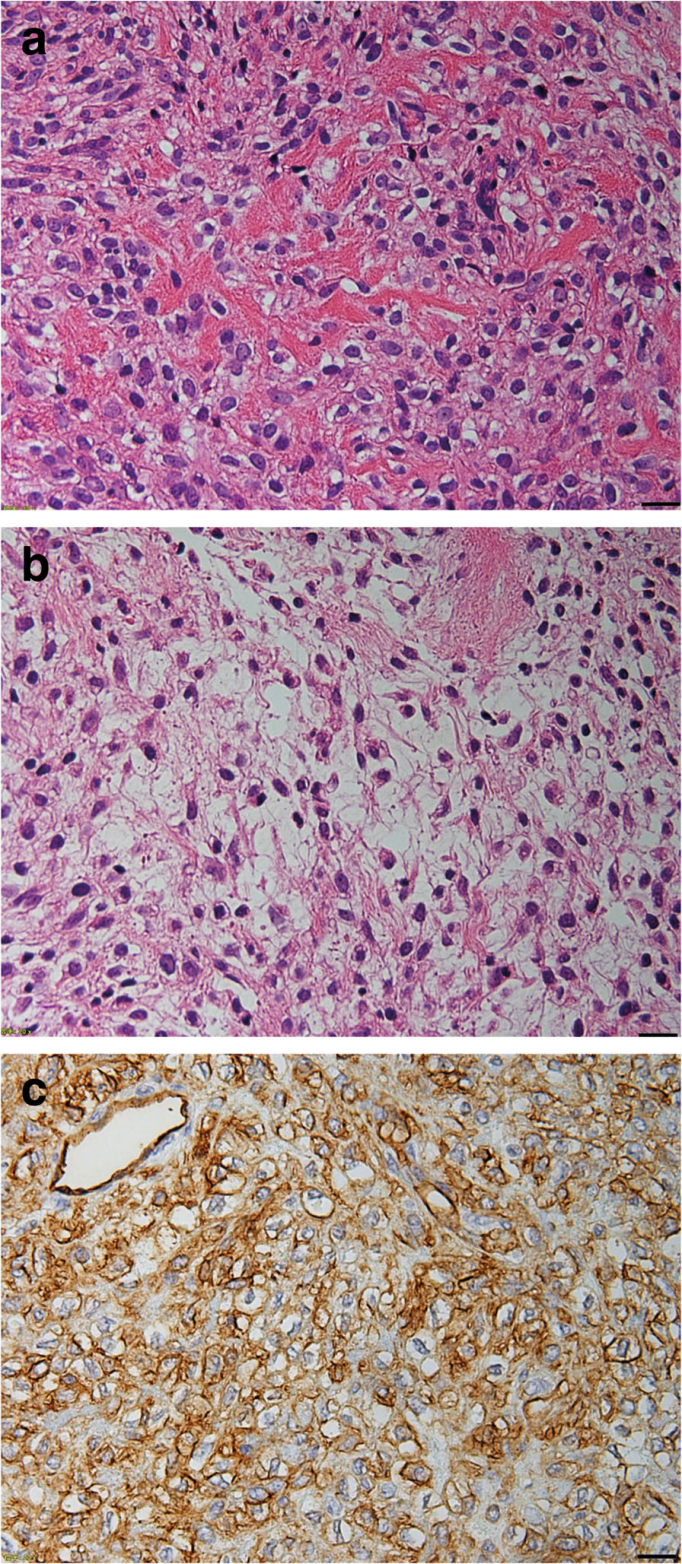
Photomicrographs of specimens collected during the first surgery. **a** Resection specimen shows a tumour comprising haphazardly proliferating high-grade spindle cells and an extracellular eosinophilic osteoid-like matrix. **b** Resection specimen shows a hypocellular area with brisk mitotic activity. Haematoxylin and eosin staining, original magnification ×400 (**a**–**b**). **c** Immunostaining of a resection specimen with primary antibodies to CD34 shows diffusely positive cells. Original magnification ×400

Haematoxylin and eosin were used for routine staining. Histologically, the tumour exhibited pleomorphic spindle cell proliferation with focal eosinophilic material (Fig. 5a). Initially, we considered the tumour extracellular matrix to be osteoid. The nuclei were spindle- or oval-shaped, and nuclear atypia was obvious. A hypocellular area with abundant mitotic activity was also observed (Fig. 5b).

Immunohistochemistry revealed that the tumour cells were positive for CD34 (Fig. 5c), CD99, and B cell lymphoma (Bcl)-2, but negative for epithelial membrane antigen, α-smooth muscle actin (αSMA), and S-100. Our immunohistochemical findings were not typical of osteosarcoma, which commonly expresses S-100 protein or αSMA. However, the observed CD99 positivity and consistent histological features led to an initial diagnosis of osteosarcoma.

### Postoperative course

The patient’s postoperative course was uneventful, and chemotherapy was initiated 1 month after surgery. Ifosfamide, doxorubicin, etoposide, cyclophosphamide, and cisplatin were discontinued 4 months after surgery. Upon discharge, the patient could ambulate with a cane.

**Fig. 6:**
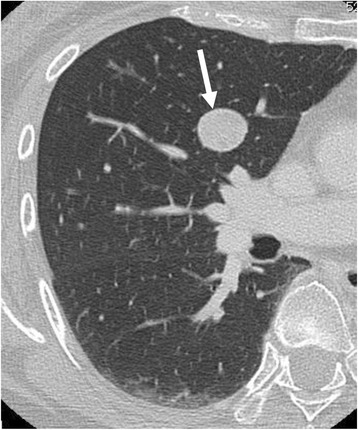
Chest computed tomography (CT) scan obtained 5 years after the first surgery. A contrast-enhanced CT scan of the chest reveals a well-defined large solitary pulmonary nodule (*arrow*) in the right middle lobe

Five years after surgery, chest CT scans showed a 20-mm nodule in the middle lobe of the right lung (Fig. 6). A second operation was performed to remove this tumour via middle lobectomy and upper lobe wedge resection. A histological examination was conducted under the suspicion that the findings would indicate a malignant SFT and not metastasis of an osteosarcoma.

### Pathological findings from a re-examination of the first surgical specimen

**Fig. 7:**
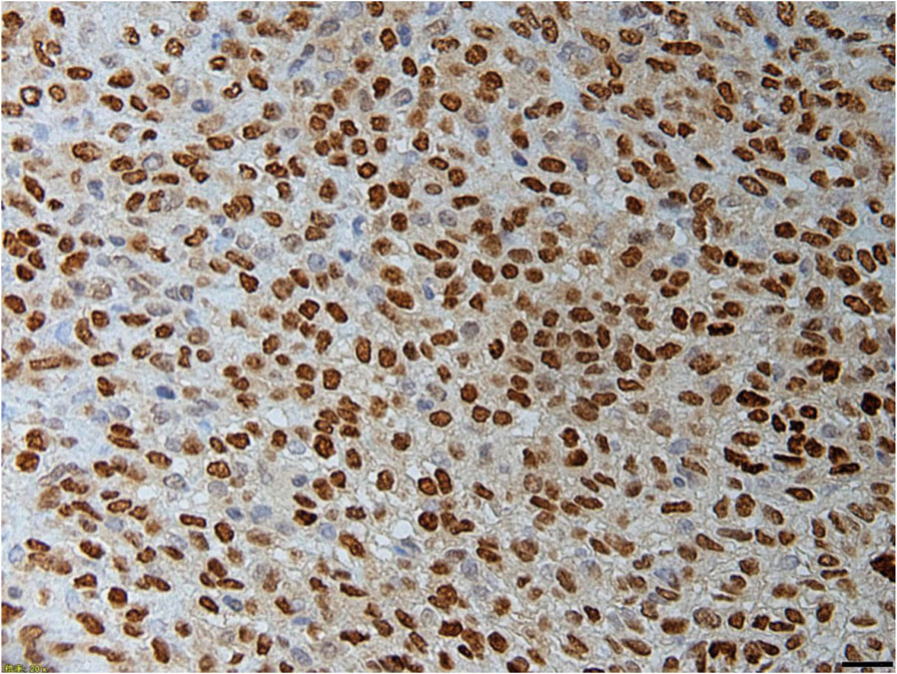
Photomicrograph of a specimen from the first surgery that was subjected to immunohistochemical re-examination. Most tumour cells exhibit nuclear expression of STAT6. Original magnification ×400

**Fig. 8:**
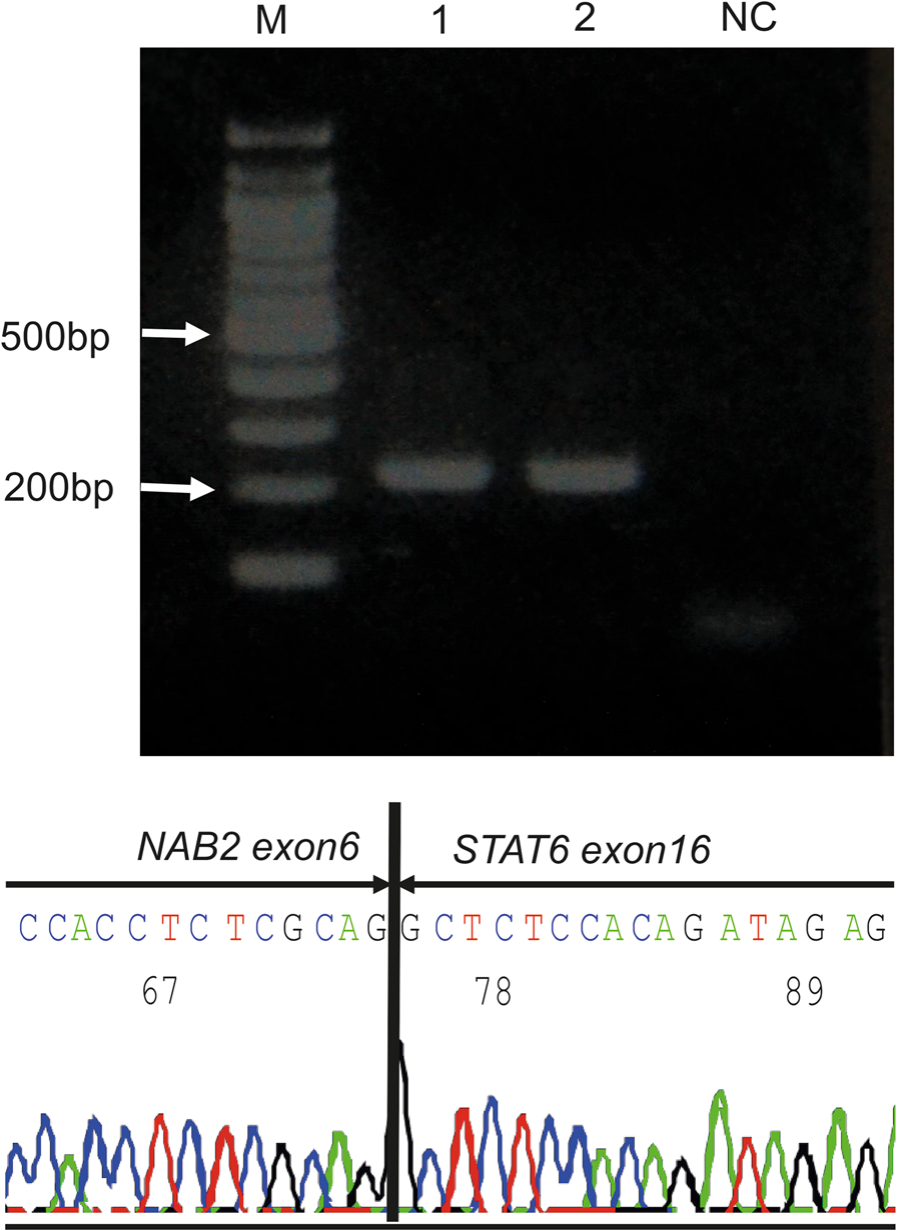
RT-PCR analysis of *NAB2-STAT6* fusion gene expression in a fresh-frozen specimen. *Upper*: PCR-amplified DNA fragments indicate a true fusion gene after sequencing. *Lane M* contains a 100-bp ladder marker. *Lanes 1* and *2* show the products of PCR with the primer pair F-NAB2 ex6 forward-2/R-STAT6 exon 17 reverse, which yielded DNA fragments of approximately 219 bp. *Lane NC* shows the result of PCR using distilled water. *Lower*: Direct sequencing analysis indicated the fusion of *NAB2 exon 6* and *STAT6 exon 16*

The first surgical specimen, which had been embedded in a paraffin block and cryopreserved, was re-examined using immunohistochemistry and reverse transcription-polymerase chain reaction (RT-PCR). The immunohistochemical analysis of paraffin-embedded sections prepared from this specimen revealed a diffuse positive STAT6 staining in the tumour cell nuclei (Fig. 7). RT-PCR detection of the *NAB2-STAT6* fusion gene was conducted using total RNA isolated from the frozen material and PCR primer sets that had been designed according to a previously reported method [5]. The forward primer, which targeted exon 6 of *NAB2* (5′-3′), was F-NAB2 ex6 forward-2, AGCAGACACTGATGGACGAG, and the reverse primer, which targeted exon 16 or 17 of *STAT6* (5′-3′), was STAT6 ex 17 reverse, TGGGCTTCTTGGGATAGAGA. These primers yielded PCR-amplified DNA fragments with approximate lengths of 219 bp (Fig. 8, upper). Direct sequencing of the PCR products detected a *NAB2-STAT6* fusion gene, *NAB2 exon 6-STAT6 exon 16* (Fig. 8, lower). Based on these findings, the final diagnosis was malignant SFT of the lumbar spine.

### Postoperative course after the second surgery

**Fig. 9:**
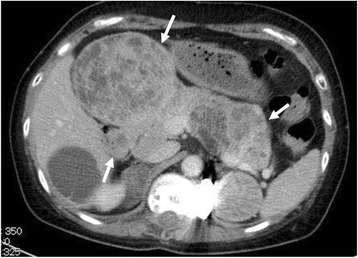
Computed tomography (CT) at 2 months after the second surgery. Axial contrast-enhanced CT reveals multiple metastatic lesions in the pancreas (*arrows*)

Systemic chemotherapy with gemcitabine and paclitaxel was initiated. However, CT scans taken 2 months after the second surgery revealed a metastatic lesion in the pancreas (Fig. 9). Although the patient had no evidence of local recurrence, she died from uncontrolled disease progression 8 years after the first surgery.

## Discussion

SFT, a rare neoplasm, was first described in 1931 by Klemperer and Rabin as a spindle cell tumour that originated from the pleura [1]. Although SFTs are not frequently observed in extrapleural locations, they have been found to arise in any location, including the spine [2–4]. Spinal SFTs usually arise within intramedullary (58%), intradural extramedullary (24%), or extradural (18%) components [6]; to the best of our knowledge, no previous reports in the scientific literature have described a malignant SFT arising from the vertebral arch.

On MRI scans, most SFTs appear hypointense or isointense relative to the muscle on T1-weighted images and have a heterogeneously hyperintense tumour component on T2-weighted images [7]. Additionally, SFTs are usually well delineated and tend to exhibit a lobulated shape with displaced adjacent structures. Local invasion is rare [8]. However, in the current case, the findings of mild tumour hyperintensity on T1-weighted images, an unclear margin, and local invasion were suggestive of malignancy.

Histologically, SFTs comprise spindle cells in a “patternless pattern” characterised by a combination of alternating hypocellular and hypercellular areas interspersed with thick bands of hyalinised collagen [9]. However, it may be difficult to distinguish collagen from other eosinophilic extracellular materials, especially osteoid, as this process is subjective [7]. Accordingly, it is extremely difficult to histologically distinguish SFTs arising in the bone from other neoplasms, especially osteosarcoma. Initially, in this case, we considered the extracellular matrix to be osteoid, leading to an initial diagnosis of osteosarcoma.

Immunohistochemical examination is useful for distinguishing SFTs from other neoplasms. Most SFTs exhibit positive staining for CD34, bcl-2, and CD99, but negative staining for αSMA, desmin, and S-100 protein. However, these immunohistochemical markers are nonspecific and can be detected in a variety of other neoplasms [10]. Recently, whole-genome sequencing was used to detect a fusion gene of *NAB2* and *STAT6* on chromosome 12 in the majority of SFTs [11]. Moreover, immunohistochemical analyses of STAT6 revealed a diffuse nuclear expression in almost all SFTs, but not in other tumours [12, 13]. Therefore, STAT6 could be considered a highly sensitive and nearly ideal marker for the differentiation of SFT from other neoplasms [13]. In this case, although the initial diagnosis was osteosarcoma, a re-examination that included immunohistochemical detection of STAT6 and RT-PCR detection of the *NAB2 exon 6-STAT6 exon 16* fusion led us to confirm a diagnosis of malignant SFT.

Although most SFTs have benign features, these tumours may recur or metastasise in the absence of any predictive morphological features [14]. As a result, SFTs are now classified as an intermediate category within the 2013 World Health Organization criteria of tumours of the soft tissue and bone [7]. Vallet-Decouvelare et al. reported that 80% of extrathoracic SFTs with atypical histological features—increased cellularity, nuclear pleomorphism, necrosis, and four or more mitoses per ten high-power fields—developed local recurrences or distant metastases [14]. In addition, Gold et al. reported that positive surgical margins and a tumour size of 10 cm or larger are prognostic factors for poor metastasis-free survival [3]. From a genetic point of view, Barthelmess et al. reported that in comparison to other types of SFTs, those harbouring *NAB2-exon 6-STAT6 exon 16/17* fusions more frequently exhibited cellular histologic features associated with more aggressive behaviour [15]. The present case, which featured infiltration, nuclear atypia, and mitosis with clinically aggressive behaviour, was ultimately diagnosed as malignant SFT.

In this case, we performed an interbody fusion using anterior, rather than posterior, instrumentation because posterior reconstruction after extensive soft tissue removal could increase the risk of postoperative infection. Fortunately, the patient’s postoperative course was uneventful, and she was immediately able to receive adjuvant chemotherapy after surgery.

We found the T-saw to be useful for cutting pedicles. The exact locations of these pedicles were difficult to identify because the posterior elements of the vertebrae were almost completely covered with extensive soft tissue, which had to be resected en bloc. It might have been dangerous to cut the pedicles from the lateral side to the medial side using an osteotome in this case, given the risk of neural damage. Although additional time was needed to set the T-saw around the pedicles, the process of cutting the pedicle from the medial wall to the lateral wall was safer.

Some authors have reported the surgical resection of SFTs arising from the spine [2, 6, 8]. Bouyer et al. reported the case of a SFT of the thoracic spine with two recurrences, and another author reported a case in which a malignant SFT was resected surgically but a local recurrence developed rapidly [8]. These cases demonstrate the difficulty associated with complete resection of a SFT arising from the spine. In our case, a total resection was achieved, and the patient had no evidence of local recurrence. However, she developed a lung metastasis 5 years after the primary resection and died of disease progression 8 years after surgery. Accordingly, a long follow-up is required because a late recurrence or metastasis can occur after more than 10 years [14].

## Conclusions

We have reported a malignant case of a SFT that occurred in the L1 vertebral arch. Malignant SFTs rarely occur in vertebral arches and are very difficult to diagnose accurately using histologic techniques. Immunohistochemical STAT6 staining is very useful for distinguishing SFTs from other neoplasms, such as osteosarcoma. Although it is difficult to completely resect a SFT arising from the spine, we have demonstrated the feasibility of an en bloc approach to the removal of primary spinal tumours arising from posterior elements without local recurrence.

## Abbreviations

CT: Computed tomography
L1: First lumbar
L3: Third lumbar
MRI: Magnetic resonance imaging
SFT: Solitary fibrous tumour
T12: 12th thoracic
αSMA: α-Smooth muscle actin
